# Time interval between the onset of connective tissue disease symptoms and first contact with a rheumatologist: results from the German National Database of collaborative arthritis centers

**DOI:** 10.1007/s00296-023-05335-0

**Published:** 2023-06-01

**Authors:** Anna Kernder, Katja Thiele, Gamal Chehab, Matthias Schneider, Johanna Callhoff

**Affiliations:** 1 Department of Rheumatology, University Hospital Düsseldorf, Medical Faculty of Heinrich Heine University, Düsseldorf, Germany; 2 Hiller Research Center, University Hospital Düsseldorf, Medical Faculty of Heinrich Heine University, 40224 Düsseldorf, Germany; 3 German Rheumatism Research Centre Berlin (DRFZ), Charitéplatz 1, 10117 Berlin (Mitte), Germany; 4 Institute of Social Medicine, Epidemiology and Health Economics, Charité-Universitätsmedizin Berlin, Charitéplatz 1, 10117 Berlin, Germany

**Keywords:** Diagnosis, Connective tissue disease, Systemic lupus erythematosus, Systemic sclerosis, Sjögrens syndrome

## Abstract

The long-term outcome of connective tissue diseases is associated with the time from symptom onset to diagnosis. To understand gaps in care, we determine whether the length of time between symptom onset and first presentation to a rheumatologist has changed in Germany in recent decades. We analyzed data on patients diagnosed with connective tissue diseases (*n* = 19,662) collected from the German National Database of the Regional Cooperative Rheumatology Centers. We reviewed the onset of relevant symptoms listed at first presentations from 1993 to 2018 and performed a quantitative analysis of the intervals until first presentation to a rheumatologist. We compared time intervals and performed a linear mixed regression model with random effects to identify associated factors. Although the interval between the onset of symptoms and first presentation to a rheumatologist has diminished since 1980 for all connective tissue diseases, there has been no relevant improvement during the past 2 decades. The interval between symptoms and presentation increases with patients age for all connective tissue diseases (e.g., Systemic sclerosis; for each 10-year-increment of patients age: β 0.41, CI 0.38; 0.44). Among those diagnosed with systemic sclerosis, the mean interval was 1.5 years (95% CI 1.1; 1.8) for male patients and 2.6 years (95% CI 2.4; 2.8) for females. Patients presenting with different degrees of disease severity on their first visits and with different educational levels had similar mean intervals between symptoms and first presentation regardless of their final diagnoses. Over the past 2 decades, the time to first consultation with a rheumatologist has not continued to improve in Germany, but has stagnated at the same level. Selected patient subgroups, such as older patients with suspected connective tissue diseases and female patients with suspected systemic sclerosis, are at risk to present late and may in particular benefit from an earlier referral to a rheumatologist.

## Introduction

Connective tissue diseases are complex disorders that may present with a wide spectrum of non-specific initial manifestations. In addition, differential diagnoses are extensive and remain challenging. These difficulties frequently result in a prolonged time interval between the onset of symptoms and diagnosis.

As time intervals between the onset of symptoms and diagnosis, 9–48 months are reported for patients with systemic lupus erythematosus (SLE) or systemic sclerosis [1–4]. Similarly, in a study of 121 SLE patients in the United Kingdom, 70% reported that they had initially received a different diagnosis and underwent 10 consultations before they were finally diagnosed [5]. However, results from recent studies revealed that most of the time elapsed between symptom onset and diagnosis of SLE occurred before the first contact with a rheumatologist [6–8].

This is a critical finding, given that diagnosis and treatment of SLE within 6 months of symptom onset results in fewer relapses, reduced rates of hospitalization, and an overall reduction in disease-associated damage [9, 10]. Likewise, the results of studies focused on systemic sclerosis also indicate that early initiation of therapy may prevent lung dysfunction and skin fibrosis [11].

Given our current understanding of the importance of early diagnosis and treatment, our study aimed to determine if the interval between symptom onset and first presentation to a rheumatologist has changed over the past decades and to identify factors that may lead to significant delays. Our findings are important to identify patient cohorts that remain in need of an earlier referral to a rheumatologist.

## Methods

We analyzed data collected from the German National Database (NDB) of the regional collaborative arthritis centers in which data from participating rheumatology practices and outpatient clinics of university hospitals as well as community hospitals were collected. Data of patients with confirmed diagnoses of inflammatory rheumatic diseases have been prospectively collected in the German National Database since 1993, and the NDB is described in more detail by Albrecht et al. [12]. We analyzed data of all patients diagnosed with connective tissue diseases, including SLE, primary Sjögren’s syndrome, systemic sclerosis, myositis, and other connective tissue disease (including undifferentiated and mixed connective tissue disease). Diagnoses were documented by the treating rheumatologists, we had no information about the diagnostic criteria. Patients or the public were not involved in the design, conduct, reporting or dissemination plans of our research.

Symptom onset was primarily collected for the NDB at the patient’s first rheumatologist visit within the NDB (reported as month and year of “Onset of typical complaints/symptoms”). Data collected in 1993–2018 were used for this analysis. Patient-reported symptom onset was used in the absence of physician-reported data. The proportion of patient data varied depending on the disease subtype between 11 and 18%. The highest proportion of patient-reported symptom onset was observed in Sjögren’s disease (18%). For the other diseases, the proportion of patients data was lower (SLE 13%, systemic sclerosis 11%, myositis 13%, other undifferentiated connective tissue diseases 12%).

We used these findings to determine the time elapsed prior to the first rheumatology presentation as stratified by the time of symptom onset. We also collected patient demographics, including age, sex, years of education, and disease severity; the latter parameter was documented by physicians for the NDB (numeric rating scale (NRS) 1–5: asymptomatic (1), mild (2), moderate (3), severe (4), very severe (5)). This information allows us to study the association of disease severity and the time between symptom onset and first presentation to a rheumatologist across all connective tissue diseases. The findings were analyzed using linear mixed regression models with the clinical center/hospital as a random variable and calculated the mean time to the first presentation to a rheumatologist by age, sex, varying disease severity and years of education. Kruskal–Wallis Tests were used to compare the diagnostic delay of different decades within the diagnostic groups. Analyses were performed with IBM SPSS version 26 and SAS version 9.4.

## Results

### Time elapsed between first symptoms and first rheumatologist contact (1980–2018)

**Fig. 1:**
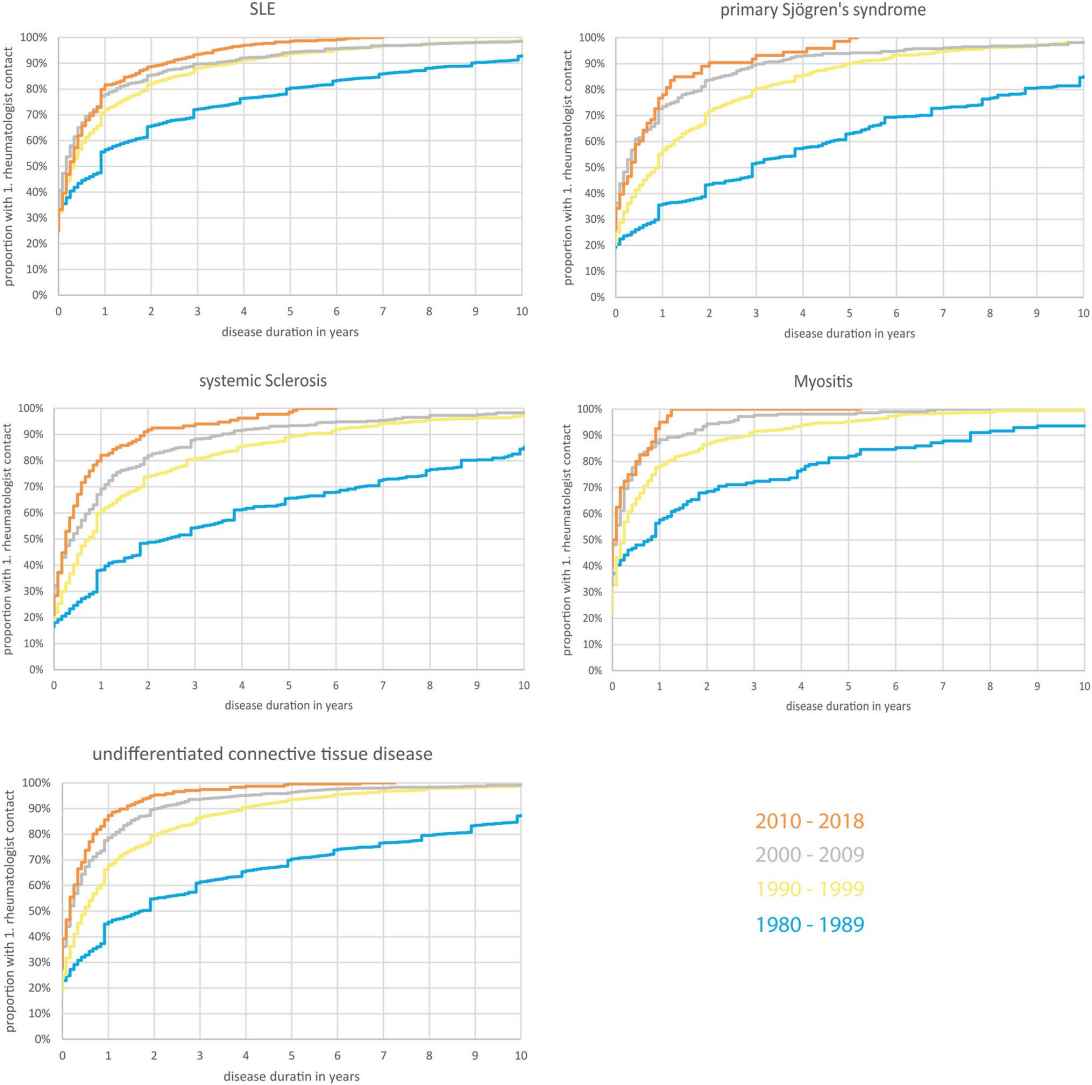
Time elapsed between onset of symptoms and first rheumatological presentation for patients diagnosed with systemic lupus erythematosus (SLE), primary Sjögren’s syndrome, systemic sclerosis, myositis, and undifferentiated connective tissue diseases

We analyzed data from a total of 19,662 records of patients diagnosed with connective tissue diseases and found that the time interval between the onset of symptoms and the first rheumatological presentation for all connective tissue diseases has diminished since 1980, although there has been no relevant improvement since 2000 (Kruskal–Wallis Test). For example, in the cohort of patients diagnosed with SLE (*n* = 8136), the median time between symptom onset to first rheumatologist contact was 3.0 years before 1980 and dropped to 0.4 years in 1990–1999. By contrast, the median time elapsed was 0.3 years from 2000 to 2009 and remained unchanged between 2010 and 2018. Additional information is provided in Table 1 and Fig. 1.

**Table 1:** Patients’ characteristics and median duration between symptom onset and first rheumatological presentation stratified by year of symptom onset

	Missing values *n*	SLE (*n* = 8136)	Primary Sjögren’s syndrome (*n* = 2362)	Systemic sclerosis (*n* = 2299)	Myositis (*n* = 1036)	Undifferentiated connective tissue disease (*n* = 5789)
Women, %	0	89	92	83	67	87
Age in years, mean (sd)	0	42 (15)	51 (14)	54 (13)	51 (15)	47 (14)
Disease severity at first visit (physician’s assessment; %)						
Asymptomatic	1433	7.3	3.7	1.9	5.0	6.8
Mild		41	48	34	27	51
Moderate		38	41	43	47	34
Severe		12	7.2	18	17	7.1
Very severe		1.9	0.4	3.9	4.1	0.7
Time symptom onset to presentation; median time in years (number of patients)	0	0.5 (*n* = 8136)	1.0 (*n* = 2362)	1.0 (*n* = 2299)	0.3 (*n* = 1036)	0.7 (*n* = 5789)
Before 1980		3.0 (*n* = 1003)	11.0 (*n* = 253)	7.0 (*n* = 216)	6.0 (*n* = 67)	9.0 (*n* = 423)
1980–1989		1.0 (*n* = 2225)	3.0 (*n* = 529)	2.5 (*n* = 463)	0.9 (*n* = 156)	1.9 (*n* = 1141)
Before 1980 vs. 1980–1989 (*p*-value)		< 0.001	< 0.001	0.008	< 0.001	< 0.001
1990–1999		0.4 (*n* = 3394)	1.0 (*n* = 1130)	0.8 (*n* = 1077)	0.3 (*n* = 559)	0.6 (*n* = 2982)
1980–1989 vs. 1990–1999 (*p*-value)		< 0.001	< 0.001	< 0.001	0.04	< 0.001
2000–2009		0.3 (*n* = 1111)	0.3 (*n* = 377)	0.5 (*n* = 409)	0.2 (*n* = 214)	0.3 (*n* = 1007)
1990–1999 vs. 2000–2009 (*p*-value)		0.04	< 0.001	< 0.001	< 0.001	< 0.001
2010–2018		0.3 (*n* = 403)	0.4 (*n* = 73)	0.3 (*n* = 134)	0.1 (*n* = 40)	0.3 (*n* = 236)
2000–2009 vs. 2010–2018 (*p*-value)		0.37	0.76	0.09	0.50	0.10

*p*-values were obtained from a Kruskal–Wallis Test

*SLE* Systemic lupus erythematosus, *sd*, disease severity 1–5 (1 = asymptomatic, 1 = mild, 2 = moderate, 3 = severe, 4 = very severe) standard deviation

### Characteristics of patients in relation to rheumatological presentation

We then examined demographic factors and determined their association with the interval between first symptoms and first rheumatology presentation (Table 2). In all connective tissue diseases, the interval between first symptoms and contact with a rheumatologist was associated with the patient’s age: For each 10-year increment of patient’s age, the presentation was ~ 0.4 years later (e.g., systemic sclerosis β 0.41, CI (0.38; 0.44)). Among those diagnosed with systemic sclerosis, the mean interval between first symptoms and diagnosis was 1.5 years (95% CI 1.1; 1.8 years) for male patients and 2.6 years (95% CI 2.4; 2.8 years) for females. Patients presenting with different degrees of disease severity on their first visits had similar mean intervals between symptoms and first presentation regardless of their final diagnoses. Similarly, we detected no correlations between educational levels and the time elapsed between symptom onset and the first rheumatology visit.

**Table 2:** Results of mixed linear regression analyses with clinic/hospital as random variable and duration from symptom onset to first contact with a rheumatologist as independent variable

	SLE estimates (95% CI)	Primary Sjögren’s syndrome estimates (95% CI)	Systemic sclerosis estimates (95% CI)	Myositis estimates (95% CI)	Undifferentiated connective tissue disease estimates (95% CI)
Age (per 10 years)	0.38 (0.36; 0.41)	0.46 (0.43; 0.49)	0.41 (0.38; 0.44)	0.19 (0.15; 0.24)	0.36 (0.34; 0.39)
Gender (female)	1.7 (1.7; 1.8)	2.5 (2.4; 2.7)	2.6 (2.4; 2.8)	1.2 (1; 1.5)	1.9 (1.8; 2)
Gender (male)	1.8 (1.5; 2)	2.3 (1.8; 2.9)	1.5 (1.1; 1.8)	1 (0.6; 1.4)	1.5 (1.2; 1.7)
Disease severity*					
Asymptomatic (1)	1.1 (0.8; 1.5)	1.4 (0.6; 2.2)	0.8 (− 0.3; 2)	0.1 (− 0.9; 1.1)	1 (0.6; 1.4)
Mild (2)	1.1 (0.9; 1.3)	1.7 (1.4; 2)	1.5 (1.2; 1.8)	0.2 (− 0.3; 0.7)	1.2 (0.9; 1.4)
Moderate (3)	0.9 (0.7; 1.1)	1.6 (1.2; 1.9)	1.6 (1.3; 1.9)	0.3 (− 0.1; 0.7)	0.9 (0.6; 1.1)
Severe (4)	0.9 (0.6; 1.2)	1.8 (1.2; 2.5)	1.1 (0.6; 1.5)	0.3 (− 0.3; 0.9)	0.9 (0.5; 1.4)
Very severe (5)	1.4 (0.7; 2)	1.5 (− 1.4; 4.4)	1.7 (0.8; 2.5)	1.2 (0; 2.4)	0.2 (− 1.1; 1.5)
Education (year)	− 0.007 (− 0.029; 0.014)	0.041 (0.017; 0.064)	0.032 (0.007; 0.057)	− 0.061 (− 0.089; − 0.032)	− 0.004 (− 0.025; 0.018)

*SLE* systemic lupus erythematosus

*Model adjusted for age and sex

## Discussion

Due to the diverse and partly non-specific initial manifestations of connective tissue diseases, the time between symptom onset and diagnosis of these diseases remains long [1, 4, 6]. Our study aimed to determine whether the interval between symptom onset and first rheumatological presentation has changed in Germany since 1980.

Our findings revealed that the interval between the onset of symptoms and the first rheumatological presentation has improved significantly for all connective tissue diseases, with reductions from several years to only a few months during the past 2 decades. However, no further improvements have been observed since 2000. Our findings are similar to those reported by Doria et al. [4] who noted that diagnosis of SLE was facilitated by the widespread introduction of anti-nuclear antibody testing around 1980 without any further improvement in the recent years. In addition, the better knowledge of autoimmunity and autoimmune diseases as well as the publication of classification criteria of connective tissue diseases (e.g., American College of Rheumatology (ACR)) probably led to a faster presentation to a rheumatologist between 1980 and 2000. Our findings revealed that the interval between symptom onset and the first rheumatological evaluation was directly associated with the patient’s age.

The non-specific initial manifestations of connective tissue diseases may be difficult to classify in older patients, particularly in those with concomitant co-morbidities. Likewise, the clinical presentation and initial manifestations of SLE change with patient’s age as patients who develop SLE at > 50 years of age typically exhibit less renal involvement, reduced complement consumption and lower levels of anti-DNA antibodies upon laboratory evaluation [13, 14].

We also determined that, among men diagnosed with systemic sclerosis, the interval between symptom onset and first rheumatological evaluation was shorter than that exhibited by women. These findings parallel those of Hudson et al. [1] who evaluated registry data describing 408 patients with systemic sclerosis and Raynaud’s syndrome. They also found that the interval between symptom onset and first presentation to a rheumatologist was shorter for men than for women. They speculated that these findings might relate to either a different biological course of the disease or different patterns of utilization of the health care system.

Interestingly, the physician’s assessment of severity of the disease at first presentation did not correlate with the time to the first presentation after symptom onset. Unfortunately, it was not possible to determine the organ involvement at the first presentation from our data.

### Limitations and strengths

After 2005, most of the patients enrolled in the National Database were followed at only five specific institutions [12], whereas before, more institutions were involved in data acquisition. This may represent a selection bias, as the currently reporting institutions may be less representative of the rheumatology health care situation in Germany. However, they do cover both inpatient and outpatient settings and offer long-standing “early arthritis clinics” that allow for short-term presentation to a rheumatologist. In addition, only patients in rheumatologic care were documented in the NDB, those who are exclusively treated by general practitioners were not included. This biases the population to include more severely affected patients than in the general patient population in Germany. As the time of symptom onset was recorded retrospectively, we do not have all relevant information on patient characteristics at symptom onset. Thus, we cannot identify all causal factors that may have a direct influence on the timing of the first rheumatology consultation (e.g., including doubts regarding a possible diagnosis by the general practitioner, lack of appointments in the centers, delay by the patients, type of disease symptom, presence of family history of rheumatic and/or autoimmune diseases). However, we were able to collect some information with longer intervals between symptom onset and the first rheumatologist visit. The strength of our analysis rests primarily on the large number of patients included in this study (*N* = 19,662) which has facilitated the comparison between specific diagnoses.

In summary, the results of our analysis revealed that older patients typically experienced a longer delay between symptom onset and presentation to a rheumatologist. By contrast, disease severity and educational level did not correlate with the length of this interval. In systemic sclerosis, we determined that male patients presented to a rheumatologist earlier after the onset of symptoms than female patients. Taken together, these results suggest that older patients with suspected connective tissue diseases and female patients with suspected systemic sclerosis are at risk to present late and may in particular benefit from an earlier referral to a rheumatologist.

## Data Availability

Data are not publicly available.
